# “Maybe if I stop the drugs, then maybe they’d care?”—hospital care experiences of people who use drugs

**DOI:** 10.1186/s12954-019-0285-7

**Published:** 2019-02-13

**Authors:** Soo Chan Carusone, Adrian Guta, Samantha Robinson, Darrell H. Tan, Curtis Cooper, Bill O’Leary, Karen de Prinse, Grant Cobb, Ross Upshur, Carol Strike

**Affiliations:** 1 Casey House, 119 Isabella St, Toronto, ON M4Y 1P2 Canada; 2 Department of Health Research Methodology, Evidence, and Impact, McMaster University, 1280 Main St W, Hamilton, ON L8S 4K1 Canada; 3 School of Social Work, University of Windsor, 167 Ferry Street, Windsor, ON N9A 0C5 Canada; 4 Dalla Lana School of Public Health, University of Toronto, 155 College St, Toronto, ON M5T 3M7 Canada; 5 St. Michael’s Hospital, 30 Bond St, Toronto, ON M5B 1W8 Canada; 6 Ottawa Hospital Research Institute, 501 Smyth Box 511, Ottawa, ON K1H 8L6 Canada; 7 Factor-Inwentash Faculty of Social Work, University of Toronto, 246 Bloor St W, Toronto, ON M5S 1V4 Canada; 8 AIDS Committee of Ottawa, 19 Main St, Ottawa, ON K1S 1A9 Canada; 9 Lunenfeld Tanenbaum Research Institute, Sinai Health System, 600 University Ave., Toronto, ON M5G 1X5 Canada

**Keywords:** Drug use, Hospital care, Qualitative research, Stigma, Patient experience

## Abstract

**Background:**

Drug use is associated with increased morbidity and mortality but people who use drugs experience significant barriers to care. Data are needed about the care experiences of people who use drugs to inform interventions and quality improvement initiatives. The objective of this study is to describe and characterize the experience of acute care for people who use drugs.

**Methods:**

We conducted a qualitative descriptive study. We recruited people with a history of active drug use at the time of an admission to an acute care hospital, who were living with HIV or hepatitis C, in Toronto and Ottawa, Canada. Data were collected in 2014 and 2015 through semi-structured interviews, audio-recorded and transcribed, and analyzed thematically.

**Results:**

Twenty-four adults (18 men, 6 women) participated. Participants predominantly recounted experiences of stigma and challenges accessing care. We present the identified themes in two overarching domains of interest: perceived effect of drug use on hospital care and impact of care experiences on future healthcare interactions. Participants described significant barriers to pain management, often resulting in inconsistent and inadequate pain management. They described various strategies to navigate access and receipt of healthcare from being “an easy patient” to self-advocacy. Negative experiences influenced their willingness to seek care, often resulting in delayed care seeking and targeting of certain hospitals.

**Conclusion:**

Drug use was experienced as a barrier at all stages of hospital care. Interventions to decrease stigma and improve our consistency and approach to pain management are necessary to improve the quality of care and care experiences of those who use drugs.

## Introduction

In 2010, mental and substance use disorders were estimated to account for 22.9% of years lived with disability (YLDs) worldwide and 7.4% of all disability-adjusted life years (DALYs) [1], with drug use disorders directly accounting for 0.8% of the total DALYs worldwide [2]. While the Global Burden of Disease Study 2015 shows an overall global improvement in health between 2005 and 2015, the disability-adjusted life years associated with drug use disorders increased by more than 20% [3]. As such, illicit drug use has been identified as an important contributor to the global burden of disease [2], with Canada among the countries with a significantly higher (than the global mean) burden. People who use drugs (PWUD) are at risk of drug-related harms (e.g., infections, overdose, and death) [4–7] and greater risk of acquiring HIV, hepatitis B, and hepatitis C and co-infections for people who inject drugs [8–10].

PWUD are more likely than others to present in the emergency department [11–14], present at a later stage of illness if living with HIV [15, 16], and be admitted to hospital [17]. However, despite their complex health challenges, they face significant barriers to accessing and receiving equitable care [18] including healthcare provider stigma, inadequate pain management, and organizational factors including requirements for abstinence. Stigma related to drug use, in terms of the internalized stigma, blame, and sense of difference felt *by* PWUD and the stigmatization and discriminatory actions *towards* PWUD, may strongly impact individual’s feelings of worth and their interactions with the general public and professionals, including those in healthcare settings [19, 20]. Consequently, stigma may impact all relevant healthcare experiences and outcomes of PWUD, directly or indirectly, whether it is identified or not. PWUD have reported poor access to healthcare, and that their care was inferior to the care received by non-users [21]. When admitted to hospital, PWUD are sometimes labelled “challenging, manipulative, drug-seeking, and demanding” by healthcare workers who are not prepared, trained, or willing to meet their needs [22–24]. People who use drugs are also more likely to be discharged against medical advice [25–28]. A recent study of people who inject drugs in Vancouver, Canada, found the hospital to be a “risk environment” wherein social and structural factors contributed to participants experiencing inadequate pain and withdrawal management, consequent drug use, and increased likelihood of discharge against medical advice [29].

There is strong evidence to support the need for improved access, care, and continuity of services for PWUD; however, little focus is placed on their lived experience of receiving hospital care. Understanding the experience of accessing, negotiating, and receiving care in the hospital environment is critical to informing interventions to improve health outcomes in PWUD and quality improvement initiatives. To address this gap, we conducted a descriptive qualitative study characterizing the experience of hospital care for PWUD living with HIV and/or hepatitis C. We focused on people living with HIV and hepatitis C (HCV) because these individuals, in addition to experiencing higher rates of substance dependency, are more frequent users of hospitals and may require complex and extended care [30–32]. This population provides the opportunity to explore the experiences of people who use drugs and who have frequent interactions with healthcare providers and health systems.

## Methods

This qualitative descriptive study [33, 34] is situated in a larger program of research about the care needs of people who use drugs during hospital admissions and the role of harm reduction [35, 36]. A purposive sampling strategy was used to recruit people living with HIV and/or HCV who self-identified as using drugs and had received in-patient hospital care in the past year. Participants were recruited from the large urban centers of Ottawa and Toronto, which have concentrations of people living with HIV and HCV, people who use drugs, and large hospital networks. The study was advertised through paper and electronic advertisements at local AIDS service organizations and an HIV specialty hospital providing care to this population, and through word of mouth. The impetus for this research study emerged through conversations with members of our research program’s community advisory groups in Toronto and Ottawa. These groups provided feedback and guidance on the project from start to finish [37]. Members of these advisory groups who met the inclusion criteria for the study were eligible to participate. The study received ethical approval from the University of Toronto HIV Research Ethics Board. Participants provided written consent and were compensated for their time ($25 CAD and return transit fare).

Interviews were conducted in-person in a private room at the community organizations where recruiting occurred. Participants were asked to complete a questionnaire with demographic measures, such as age, race/ethnicity, and gender, as well as current housing, perceived physical and mental health status, drugs consumed over their lifetime and within the previous 3 months, and to rate their most recent experience receiving care in hospital. Participants were interviewed using semi-structured guides which explored their in-hospital experiences including key issues related to accessing care, the strategies used to negotiate/manage their drug use in an in-patient setting, how they perceived drug use to influence the provision of care and therapeutic relationships, and what policies and practices might ensure care is appropriate to their needs. Interviews lasted between 10 min and 1 h and were conducted between December 2014 and May 2015.

### Analysis

All interviews were audio-recorded, anonymized, and transcribed verbatim. We verified and uploaded all interview transcripts to NVivo 11 qualitative data management software. We used a thematic analysis approach, using a primarily realist perspective [38] to describe and explore the hospital experiences of this population, with the ultimate goal of understanding what components of hospital care “work”, and why, for engaging PWUD in healthcare to ultimately improve their health [39]. Following initial coding, we moved towards establishing the relationship between codes and identifying themes. We used several strategies to enhance analytical rigor. Themes were reviewed and discussed in team meetings (AG, SCC, CS). We conducted member checks with our community advisory committees to ensure our interpretation resonated and to plan next stages in the research [40]. We entered all questionnaire data into Excel and report results for categorical variables with frequencies and proportions.

## Results

We conducted 24 interviews with PWUD and living with HIV and/or hepatitis C in Ottawa (*n* = 12) and Toronto (*n* = 12), Canada. Participant characteristics are summarized in Table 1. All but two participants were living with HIV; 11 (46%) were co-infected with HIV and HCV. Twelve participants (50%) had some form of paid employment in the last 12 months. The most common drugs used in the last 3 months, other than cannabis (83%), were cocaine (62%), non-prescribed use of prescription opioids (50%), non-prescribed use of sedatives (38%), and methamphetamines (33%).

**Table 1:** Participant characteristics

Characteristic	
Gender	
Female	6 (25%)
Male	18 (75%)
Age (median, range)	48 years (33–56)
Housing	
In apartment or house they own/rent	17 (71%)
Supportive or transitional housing	6 (25%)
Homeless	1 (4%)
Ethnicity	
White/Caucasian only	18 (75%)
Aboriginal	5 (21%)
Mixed	1 (4%)
Paid employment (of any kind): last 12 months	
Yes	12 (50%)
No	11 (46%)
Permanently unable to work	1 (4%)
HIV/hepatitis C (HCV)	
HIV positive	22 (92%)
HCV positive	13 (54%)
HIV and HCV co-infected	11 (46%)
Self-reported health status	
Poor	2 (8%)
Fair	5 (21%)
Good	8 (33%)
Very Good	9 (38%)
Excellent	0
Most common substances used: last 3 months, ever	
Alcohol	19 (79%), 23 (96%)
Cannabis	20 (83%), 24 (100%)
Cocaine	15 (62%), 22 (92%)
Prescription opioids (not prescribed)	12 (50%), 20 (83%)
Sedatives	9 (38%), 18 (75%)
Methamphetamine	8 (33%), 20 (83%)
Street opioids (e.g., heroin, opium)	5 (21%), 15 (62%)
Prescription stimulants	4 (17%), 13 (54%)
Poly-substance use^a^ in last 3 months	16 (67%)

^a^Poly-substance use defined as the use of more than one drug (excluding alcohol and cannabis)

Participants described diverse experiences of care. They often stated that their experiences were not all bad; however, probes regarding positive experiences did not yield specific details and usually suggested or explicitly stated that “good” experiences were defined as a lack of negative experiences. For example, when one participant was asked why he defined his last admission as relatively good, he responded “Nobody was criticizing me or giving me a hard time.” (P-41). Participants focused most predominantly on instances of stigma and challenges accessing adequate and appropriate care, which they predominantly saw as manifestations of stigma associated with presumed drug use. We present our findings in two overarching domains of interest: perceived effect of drug use on hospital care and impact of care experiences on future healthcare interactions. These topics are not distinct. The first focuses generally on the care experience from the point of initial presentation to the time of hospital discharge during which the experience of accessing and receiving pain management emerged as an especially prominent theme. The second domain captures the impact and response of the participants to the care received, addressing the dynamic nature of engaging in care in hospital and the recursivity and impact on health seeking in the future.

### Perceived effect of drug use on hospital care

Participants did not identify all hospital experiences as negative, but clearly identified instances where drug use had negatively impacted their care experiences at all stages of the process, from deciding to seek care through to discharge from hospital. They felt drug use, or presumed drug use, strongly influenced the attitudes and relationships with clinicians, access and timeliness of care, and physicians’ adjudication and resulting offers to prescribe certain medications, most importantly those for pain management.

#### Feelings of stigma and discrimination by clinicians

Participants spoke about the stigma they felt and the discrimination they experienced from clinicians. Stigma was felt to influence how hospital staff engaged with, and spoke to, patients when drug use was known or suspected, and participants identified negative experiences as examples of discrimination.

In general, they tend to be a little stricter with you. They tend to be more short tempered with you. They tend to presume what you need, instead of a conversation, right? … And they do not actually get to know the person. (P-22)

…they have no respect for you, do not know what to do with you and do not really want to bother. (P-41)

Like, it’s a stereotype, the way some of the doctors and nurses will treat you. They have their own diagnosis of you, and if there’s drugs involved, your diagnosis is done. They do not need to look further, that’s it; that’s all. (P-42)

Participants recounted clinicians speaking negatively about them directly and to other staff or patients.

The doctor …did not know that I understood French. Right? So he says to the nurse that he’s tired of work[ing] with these type of junkie people. He wished that they did not even come to the hospital. (P-15)

I know of dozens of doctors who are terrific, but I know probably tenfold more doctors that are mean and judgmental, and negligent, and it’s not just physicians. You know, I had a nurse in the [hospital] the first week or two after my surgery that was reaming me out because I was an alcoholic and I was taking up a bed, because that’s how I got my cancer. (P-23)

One woman recounted a particularly negative experience. She presented in the emergency department (ED) to seek care following a drug deal which became violent:

And the nurse, I call her nurse Ratchet. She was horrible. She said to me, if I do not submit to this rape kit thing, that I should just leave and this bed be given to someone who needs it. And it was wintertime, and I had one shoe, and bare feet [in] the other foot. And I did not have a coat, and my clothes were torn. And you know, I left the hospital and she was just such a horrible… nurse. (P-19)

#### Challenges accessing care and timeliness of care

Participants felt that clinicians’ awareness of their past or current drug use, and associated stigmas, not only impacted how clinicians spoke and interacted with them (as discussed above) but negatively impacted the attentiveness and timeliness of their care in the ED and once admitted. Although individual events in EDs are described below, they reflect a common theme across participant experiences: perceived stigmatizing and discriminatory behavior of staff including delayed assessment and treatment. One participant described an experience in the ED with his partner: “…we were treated completely like ‘Put them over there in the corner. They’re drug users.’ And so that was, like, zero respect.” He explained that it was later discovered his partner had pneumonia and was in hospital for over 3 weeks. He spoke about his frustration and efforts to get attention:

They put us in a room way far at the back (laugh) of the [ED]. And, we did not see anybody. He actually stopped breathing a couple of times, and I ran out and I said …. ‘If anybody cares, the drug addict in the back here, may be dying.’ Cause I was just so incensed. …the second time, security did come and say ‘If this happens again, we are going to have to ask you to leave.’ (P-38)

Another participant described his fear of an ED, associated with delayed attention.

… I’d be afraid to go [to a specific hospital] if I could not breathe with my asthma. They’ll think it was something else, I do not know, and I’d probably die, so … I think they suspect that people are drug seeking right? And then they do not bother seeing if you are okay. (P-27)

This participant recounted a story of sneaking out of a back-assessment room of the ED and taking a cab to another hospital “Mainly because I was afraid I was going to die, right? So they just left me in a room, and I was like, ‘I’m out of here. So out of here.’” (P-27)

One participant recounted going to the ED with a ruptured spleen and how he had to scream in pain for some time before he was seen, identifying this as discrimination because of presumed drug dependence: “it was pretty clear to me that they really did think, you know, ‘Oh, it’s just, oh, just looking to get some painkillers.’” (P-29)

In addition to challenges faced in the ED, some participants believed that once admitted their needs were addressed more slowly than those of fellow patients.

They treat you differently because you are, when you are on the HIV ward, they treat you differently. And you are a drug user, it’s like, they forget about your meds, they are later with you or run to the other patients before they run to you. (P-26)

This participant spoke about how she used the lack of attention to her “advantage” in one of her admissions:

And being at [name of hospital], it’s easy to get away with smoking. Like, I was able to do my drugs, in my room and not get in trouble, so obviously, they were not paying attention to what I was doing. (P-26)

Being ignored had more serious consequences for some participants including the following participant who described the lack of support he received in hospital after overdosing on fentanyl. He was encouraged to go to the hospital but then “when I got there, it’s like, you know, there’s nobody’s treating me or nothing or anything. Like, so, what am I doing here?… It’s like, they didn’t want to help me anyway…” so he left, and overdosed again, this time resulting in a coma:

… cause of the Narcan that they gave, right, I felt like, a dope sickness. Right? So I went and did another fentanyl patch and that was the last thing I remember. And I remember waking up, like, [weeks] later. It was like, quite the experience, you know? (P-33)

#### Prescribing practices and pain management

Participants felt that their known or suspected drug use strongly impacted physicians’ adjudication for medications and prescribing practices.

Well, they were very cautious about prescribing me anything like Clonazepam or …Oxycontin, because ‘Oh, you are a drug user. We cannot give you that. You might get addicted to it.’ (P-31)

The stigma of being on methadone, you are treated like you are a junkie from the back alleys …So, they do not want to prescribe you anything, or that they make the assumption that you are going to sell it. (P-32)

Participants described significant barriers to pain management. In most instances this resulted in inconsistent and inadequate pain management. One participant spoke about being transferred between hospitals and the challenges she faced maintaining her pain management:

…it was hell getting my pain meds. They were like ‘You’re on forty-five milligrams three times a day? I find that hard to believe.’ […] Once I got given to a surgeon, he was like ‘This is ridiculous. Give this woman some pain medication.’ (P-21)

Participants spoke about choosing to go to one hospital over another because of discrepancies in their experiences (by facility).

I have severe Crohn’s, which is very painful. And, I went in one night, they would not give me painkillers, because I had a red flag on my chart. (P-36)

But, he identified no problems getting pain medication at another hospital where “they know how painful it was… so they know when I go in there, I’m not playing around.”

When adequate pain management was achieved, it was often after significant delay and following strong advocacy efforts, usually by the patients themselves.

Participants felt pain management decisions were not appropriately informed, particularly for those on methadone. They felt decisions were based on presumed drug use and not appropriately informed by clinical acuity, present drug use, drug of choice, or tolerance. The participant below also refers to the issue of inconsistency:

But the hospitals are normally pretty good. But once in a while, you’ll run into, you know, certain doctors there, and they do not want to do nothing for you. Like, I had my jaw busted, and I wanted something to cut through my methadone for pain, right? And I said ‘Well, I am not going to go through with the operation unless you are going to give me something for the pain.’ Like, it’s only something for, while I am in the hospital... And they did not, they did not give me nothing for it. (P-33)

This participant went through with the surgery, without the requested pain medication but later had his girlfriend bring him morphine to address his post-surgical pain.

Another participant recounts her difficulty getting pain medication because of the presumed impact of methadone and the lack of consideration for her tolerance, despite open conversations.

I am on methadone too, so I can see the difference there as well, right? Because when you are on methadone, doctors sometimes do not want to give you any kind of pain relief, even though you could probably take more pain relief than the average person. So I often have to take a letter with me, right? But I bet you if I went to [name of hospital] with that, they would not give me anything. (P-27).

Participants spoke about various approaches for dealing with the challenges of pain management while in hospital. One participant identified a common approach: *“You’ve got the power to relieve my pain; I’m going to kiss your ass.”* But she states that *“I don’t do that”*. Instead, she describes her approach: *“I told them ‘I’m not up here to jack up your emergency room to give me more opiates. I have my own pain meds. But you gotta let me take them if you’re not going to prescribe them.’ Right?”* (P-21). Other approaches included using non-prescribed medications, leaving against medical advice, and strong self-advocacy efforts to negotiate with providers for timely care and adequate pain medication. These are discussed in more detail in the final section.

### Impact of care experiences

The negative hospital experiences participants described impacted their strategies for navigating healthcare and their future health seeking.

#### Strategies for navigating hospital care

Different strategies to navigate care were apparent, as illustrated in the quotes above. Some participants spoke about trying to be a “good patient”, trying to cause as little disruption as possible.

I have had no issues so far. they have been all pretty good. I do not require a lot of medication, or I do not demand a lot… (P-40)

This participant referred to himself as *“an easy patient”*, not asking *“too many questions”* because *“you don’t want subpar treatment ever, so.”* Another participant described being a *“good patient”* and said he would *“go with the flow”* and avoid arguing with healthcare providers *“cause I know, even if they are rude, I’ll wait it out until I get what I need first. Cause if you don’t, you don’t get what you need”* (P-16). Being an easy patient was a strategy informed by past experiences and used to pave the way for future interactions:

I wanted to get high the whole time. … it was just, lack of it [drugs] being there, and I knew that I’d probably end up there [in hospital] again. So you do not want to screw with them too much, where you know, you are going to be [on] file then... I try my best to behave as much as I can. (P-34)

However, others directly confronted clinicians and used their knowledge of the healthcare system to demand care they perceived to be their right.

Okay, I have got to tell you, I do not think I have had a hideous experience in a general hospital. A), I am articulate and know what’s going on. People get bulldozed when they do not say anything. I am too emotional for that. And I cannot let people walk all over me. That does not fly with me. You know? And unfortunately, doctors think they can walk over everybody, and paint everybody with the same brush. When you are dealing with drugs, you cannot do that. (P-21)

With repeated experience in hospital, participants spoke about learning strategies to navigate the healthcare system. One participant indicated that his care changed when he spoke up: *“Oh, I mean, I got to the point where I was in the hospital, I told them, I said ‘Either you look after it, or I’m going to get up and I’m going to go out on the street; I’m going to go get my own damn drugs’. And I’m going to look after myself”* (P-29). This participant indicated that this was a learned behavior that had been successful on many occasions where with *“…such a fuss and so on, that they finally broke down and actually dealt with me. But the point is, do you have to go that far, just to get attended to? I mean, it’s ridiculous that you have to fight that hard.”*

#### Future health seeking

Negative hospital experiences influenced participants’ willingness to navigate services and timeliness of presenting for care with subsequent health issues. One participant recounted why they delayed seeking care in a timely fashion:

I was afraid. I was afraid of, they [hospital staff] would not accept me or they would not like, ‘Oh well, they could tell I am on drugs. they are not going to help me.’ … So I did not go. (P-35)

The act of seeking care when it felt necessary was often delayed when participants were directly under the influence of drugs and feared being judged. As one participant said, *“I was just too stoned, and too paranoid to leave the apartment, to actually even call 911 or go to the hospital. And too ashamed.”* (P-22). However, this delay in seeking care was further accentuated by past negative experiences.

I wanted to finish doing my drugs before I went to the hospital. (laugh) And then I did not end up going and then that left me sicker. You know? But I did not want the nightmare of [name of hospital]. Right? … It was so powerful, that little threat about no pain meds, once, that they did, that affected my outlook for [name of hospital]. Period. (P-21)

Past experiences influenced what participants were willing to disclose to clinicians but also what care they “bothered” to seek.

You want to be free to tell but you are also afraid to tell them, because then you know they are going to cut you down … you do not want to tell them that ‘Oh yeah, I smoke crack.’ or ‘I do this and I do that.’ because immediately you are screwed. Oh, now you are reduced to Percocets instead. But I need morphine. I do not need Percocets. I need morphine. But try to tell them that, right? And this is what happens. (P-29)

When we asked one participant about how they got pain medication when admitted to hospital, they responded, *“I don’t even ask… I’ve tried I don’t know how many times to get, ah, through my own family doctor, walk ins, et cetera, over the years. I don’t even, I don’t even bother. I just buy them.”* (P-15)

In some cases, lack of pain medication and poor care led to participants leaving against medical advice. One participant recounted how he was in withdrawal and they refused to give him anything to manage his pain so he “… told them ‘I’m leaving.’ Because I had my [percocets] at home, right?” (which he clarified as “stuff I bought off the street”). He added: “I was scared, because I wasn’t sure whether I should really be doing this [leaving ‘against medical advice’]. But my whole body just ached and the ibuprofen wasn’t doing anything for me.” (P-39)

## Discussion

In this study, we captured in-patient hospital experiences of PWUD living with HIV and/or hepatitis C. While not all experiences were bad, participants described clinicians’ stigma and challenges with achieving access to treatment, including insufficient and unpredictable pain management, which resulted in distrust, use of non-prescribed drugs during admission, decisions to leave against medical advice and delays and anxiety when seeking care in the future. Participants identified behavioral strategies for navigating healthcare and described choosing which hospital to present at based on past experiences.

This study brings depth to our understanding of the patient experience of hospital care for a marginalized population that is associated with frequent and high-cost health system use. Previous research has demonstrated healthcare provider stigma related to drug use and people who use drugs; however, this is most frequently captured from the provider perspective. We identify broad-ranging impacts of drug use, from the patient perspective, on negotiating, navigating and receiving hospital-based care and its recursivity. This study highlights the influence of drug use on the experience of hospital care and engagement in healthcare more broadly.

A narrative review of 158 primary research studies identified stigma towards drug users to be common among the general public and non-specialist professionals. It concluded that the stigmatization has a profound impact on the lives of PWUD including their chances of recovery [19]. A common theme from clinicians was difficult, and in some cases verbally and physically violent, interactions with PWUD. This, coupled with limited education focused on drug use and addiction in clinician training more broadly, likely has a role in the stigmatizing and negative interactions reported by people who use drugs. The review suggested that blaming and stigmatizing behaviors by clinicians “is preventing” people who use drugs “from seeking help for general health problems.” Our findings support this hypothesis, as participants described the impact of negative experiences with healthcare on all stages of accessing and engaging in care.

Hospital-based pain management for PWUD is an identified challenge for both providers and patients [41–44]. Not all experiences are, as expected, negative, and many PWUD do not report having had challenges with accessing pain management [42]; however, pain management is associated with significant distrust for both providers and patients [45, 46]. Our work supports the work of others in identifying the impact of delayed and inadequate pain management on clinician-patient interactions, decisions to use illicit drugs in hospital [47], decisions to leave hospital before treatment is complete [48], and decisions to seek care. For many in this study, the process of accessing, or being denied, pain management had an overriding impact on the experience of hospital care and decisions regarding engaging in hospital care in the present and the future.

While we intended to focus on recent in-patient hospital experiences, it became apparent that the experiences in the ED, as the place where patients present and are assessed before being admitted, and previous hospital experiences, were critical to our understanding of the in-patient care experience and, thus also, in our opinion, essential for informing strategies to improve care. Individuals’ decisions to present in the ED and negotiate accessing care, and the process of admission, were required steps in the journey of acute care and set the stage for participants’ experiences of care during an admission. Our work echoes calls for greater attention and support for addiction service provision in emergency departments [49–51]. Future research and interventions to improve the health and care of PWUDs should explore and consider not just experiences of accessing and receiving care, but also conceptions of healthcare, health seeking behaviors, and dynamic relationships within healthcare for PWUDs. Our findings also support calls for integrated care, where multiple services are provided in the same facility, enabling a more accessible and comprehensive approach to complex patients [52, 53]. Additional research and interventions may benefit by strengthening hospital-community relationships (see for example [54, 55]), impacting, on a systems level, the required steps and work necessary for PWUDs to navigate and initially present for healthcare. Interventions may include opportunities to move care out of the hospital, where possible, as one approach in a comprehensive strategy to address structural barriers.

This study has limitations that should inform its interpretation and next steps. Firstly, we recruited individuals who self-identified as using drugs from two urban settings in Ontario, Canada. The social, political, and structural context greatly influence all stages of accessing and receiving care and the impact of drug use and should be considered when exploring the applicability of these findings to other contexts. Even in these same cities, the context of the current opioid crisis has likely directly and indirectly influenced patient experiences with emergency medical services and emergency departments (since this study) for those who use, or are presumed to use, drugs. However, there is no evidence to suggest that the issues identified here are any less significant, but addressing them may be more pressing in light of increasing numbers of overdoses and hospitalizations related to opioid-related harms [56, 57]. We also focused on PWUDs who were also living with HIV and/or HCV. The demographics of our study population, while relatively representative (in terms of age and gender distribution) of people living with HIV in the province, did not capture the experiences of young adults and only 25% of our participants were women. This group of adults living with HIV/HCV likely represents a more complex and high-intensity healthcare using population of PWUDs. Interestingly, descriptions and understandings of their experiences primarily related to substance use and did not generally relate to HIV- and HCV-specific issues or conditions suggesting the themes may be relevant to the broader population of PWUD. It is also important to highlight that inclusion for this study required identification of the use of any illicit substance or the use of opioids without a prescription (the majority of participants reported poly-substance use); we did not select by, or capture, routes of administration. We were unable to explore the impact of drug use on hospital care experience by type, frequency, and patterns of drug use but expect that there are important relationships to consider.

In conclusion, this study captures the impact of drug use on hospital care from the experiences of patients. Improving the care experiences of people who use drugs may significantly improve their engagement in care and illness management [54, 58, 59]. Our findings illustrate that drug use can impact all stages of hospital care, identifying the need for it, seeking, negotiating, and engaging in it. This understanding can be helpful for both expanding and focusing our attention to the various essential steps in accessing and receiving appropriate healthcare for PWUD. We must address the issues of stigma and inconsistency of access to care, and recognize the resulting work and strategies adopted by patients to navigate the system. Perhaps most importantly, we must improve our consistency and approach to pain management for people who use drugs and understand the inter-connections between negotiating pain management and engaging in other healthcare during a hospital admission and in the future.
